# 2-Chloro-6,6-dimethyl-5,6-dihydro­indazolo[2,3-*c*]quinazoline

**DOI:** 10.1107/S1600536810003818

**Published:** 2010-02-06

**Authors:** Núbia Boechat, Adriana dos Santos Lages, Warner B. Kover, Edward R. T. Tiekink, James L. Wardell, Solange M. S. V. Wardell

**Affiliations:** a Fundação Oswaldo Cruz, Instituto de Tecnologia em Fármacos, Departamento de Síntese Orgânica, Manguinhos, CEP 21041250 Rio de Janeiro, RJ, Brazil; b Universidade Federal do Rio de Janeiro, Departamento de Química Orgânica, Instituto de Quıímica, Cidade Universitária, 21949-900 Rio de Janeiro, RJ, Brazil; c Department of Chemistry, University of Malaya, 50603 Kuala Lumpur, Malaysia; d Centro de Desenvolvimento Tecnológico em Saúde (CDTS), Fundação Oswaldo Cruz (FIOCRUZ), Casa Amarela, Campus de Manguinhos, Av. Brasil 4365, 21040-900 Rio de Janeiro, RJ, Brazil; e CHEMSOL, 1 Harcourt Road, Aberdeen AB15 5NY, Scotland

## Abstract

Two independent but virtually identical mol­ecules comprise the asymmetric unit of the title compound, C_16_H_14_ClN_3_. The mol­ecules have a slightly curved shape owing to puckering in the six-membered C_4_N_2_ ring; the respective dihedral angles formed between the benzene rings are 12.64 (7) and 11.72 (7)°. In the crystal, layers sustained by a combination of N—H⋯N hydrogen bonding as well as C—H⋯N and C—H⋯π contacts are formed; these stack along [011] and are connected by further C—H⋯π contacts.

## Related literature

For background to the synthesis and biological activity of the title compound, see: Rousselet *et al.* (1993 ▶); Ferreira *et al.* (2007 ▶). For additional geometric analysis, see Cremer & Pople (1975 ▶).
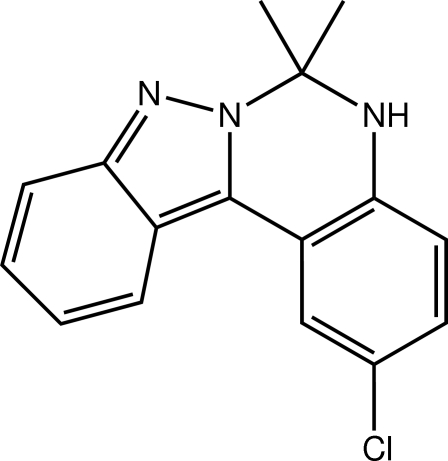


## Experimental

### 

#### Crystal data

C_16_H_14_ClN_3_
*M* *_r_* = 283.75
Triclinic, 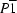

*a* = 9.8636 (2) Å
*b* = 10.7971 (2) Å
*c* = 13.2387 (3) Å
α = 93.483 (1)°
β = 100.391 (1)°
γ = 104.419 (1)°
*V* = 1334.81 (5) Å^3^
*Z* = 4
Mo *K*α radiation
μ = 0.28 mm^−1^
*T* = 120 K
0.55 × 0.25 × 0.15 mm

#### Data collection

Nonius KappaCCD area-detector diffractometer
Absorption correction: multi-scan (*SADABS*; Sheldrick, 2007 ▶) *T*_min_ = 0.885, *T*_max_ = 1.000
27218 measured reflections
6102 independent reflections
5108 reflections with *I* > 2σ(*I*)
*R*_int_ = 0.037

#### Refinement

*R*[*F* ^2^ > 2σ(*F* ^2^)] = 0.036
*wR*(*F* ^2^) = 0.094
*S* = 1.02
6102 reflections
371 parameters
H atoms treated by a mixture of independent and constrained refinement
Δρ_max_ = 0.25 e Å^−3^
Δρ_min_ = −0.37 e Å^−3^

### 

Data collection: *COLLECT* (Hooft, 1998 ▶); cell refinement: *DENZO* (Otwinowski & Minor, 1997 ▶) and *COLLECT*; data reduction: *DENZO* and *COLLECT*; program(s) used to solve structure: *SHELXS97* (Sheldrick, 2008 ▶); program(s) used to refine structure: *SHELXL97* (Sheldrick, 2008 ▶); molecular graphics: *ORTEP-3* (Farrugia, 1997 ▶) and *DIAMOND* (Brandenburg, 2006 ▶); software used to prepare material for publication: *PLATON* (Spek, 2003 ▶) and *publCIF* (Westrip, 2010 ▶).

## Supplementary Material

Crystal structure: contains datablocks global, I. DOI: 10.1107/S1600536810003818/hb5323sup1.cif

Structure factors: contains datablocks I. DOI: 10.1107/S1600536810003818/hb5323Isup2.hkl

Additional supplementary materials:  crystallographic information; 3D view; checkCIF report

## Figures and Tables

**Table 1:** Hydrogen-bond geometry (Å, °) *Cg*1, *Cg*2, *Cg*3 and *Cg*4 are the centroids of the N2,N3,C10,C11,C16, N5,N6,C26,C27,C32, C1–C6 and C17–C22 rings, respectively.

*D*—H⋯*A*	*D*—H	H⋯*A*	*D*⋯*A*	*D*—H⋯*A*
N1—H1n⋯N6	0.90 (2)	2.32 (2)	3.2090 (17)	173 (2)
N4—H4n⋯N3^i^	0.86 (2)	2.39 (2)	3.2384 (17)	168 (2)
C9—H9b⋯N4^ii^	0.98	2.58	3.537 (2)	164
C25—H25b⋯N1	0.98	2.61	3.545 (2)	160
C24—H24c⋯*Cg*1^i^	0.98	2.90	3.8431 (17)	162
C8—H8c⋯*Cg*2	0.98	2.97	3.8929 (17)	157
C18—H18⋯*Cg*3^iii^	0.95	2.92	3.6630 (15)	135
C14—H14⋯*Cg*4^iv^	0.95	2.95	3.8062 (16)	151

Symmetry codes: (i) 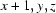; (ii) 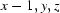; (iii) 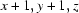; (iv) 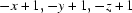.

**Fig. 1.**
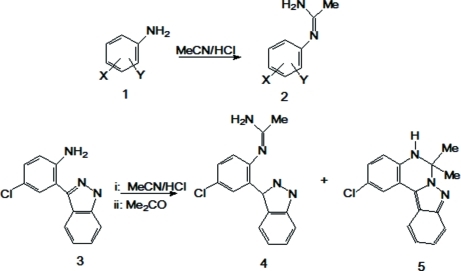
Reaction scheme for the synthesis of (I).

**Fig. 2.**
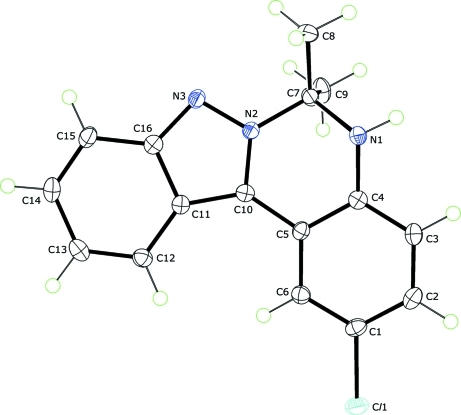
The molecular structure of the first independent molecule in (I) showing displacement ellipsoids at the 50% probability level.

**Fig. 3.**
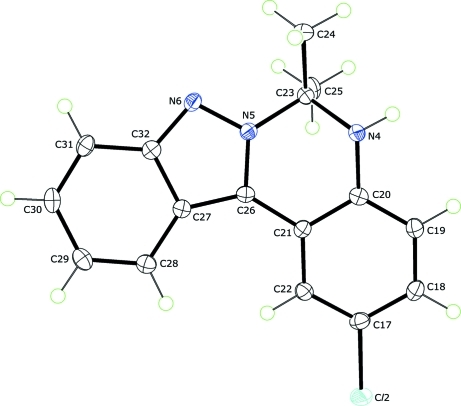
The molecular structure of the second independent molecule in (I) showing displacement ellipsoids at the 50% probability level.

**Fig. 4.**
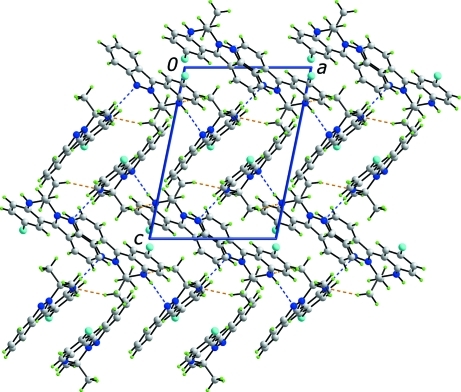
A view of the supramolecular array in (I) held together by N–H···N hydrogen bonds (blue dashed bonds), as well as C–H···N (orange dashed lines) and C–H···π (not shown) interactions. Colour code: Cl, cyan; N, blue; C, grey; and H, green.

**Fig. 5.**
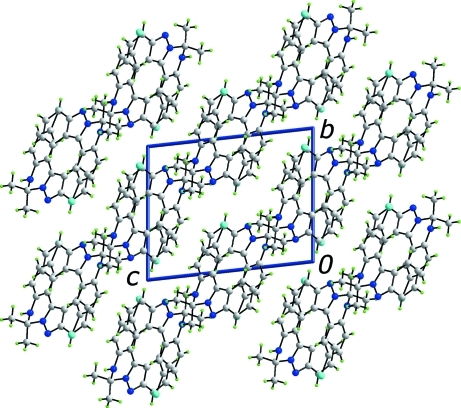
View of the stacking of layers in (I) in projection down the a axis. Colour code: Cl, cyan; N, blue; C, grey; and H, green.

## supplementary crystallographic information

### Comment

Referring to Fig. 1, amidines, 2 (e.g., X = Y = H; X = H, Y = 4-Cl, 4-Br, 4-F, 4-NO_2_, 4-CF_3_, 4-CN, 4-CH_3_, 4-OMe, 2-Me, 3-OCF_3_; X, Y = 2,6-F) can be formed from reaction of anilines, 1, with acetonitrile and gaseous hydrogen chloride (Rousselet *et al.*, 1993; Ferreira *et al.*, 2007). From, the *N*-aryl-amidines, 2, on successively reactions with 2-bromomalonaldehyde and *N*,*N*-diethylaminosulfur trifluoride (DAST), can be formed 1-(substituted-phenyl)-5-(difluoromethyl)-2-methyl-1*H*-imidazoles, potential anti-leishmanial agents (Ferreira *et al.*, 2007). Unexpectedly, the reaction of aniline 3 with acetonitrile and gaseous hydrogen chloride, followed by a workup using Me_2_CO, not only produced the amidine, 4, but also the title compound, (5 in Fig. 1 but hereafter, I). Compound (I) had been formed by a condensation reaction between the acetone and the starting material 3. The molecular and crystal structures of (I) are now reported.

Two independent but similar molecules, molecule *a* (Fig. 1) and molecule *b* (Fig. 2), comprise the crystallographic asymmetric unit in (I). The r.m.s. values for bond distances and angles are 0.0028 Å and 0.325 °, respectively. The six-membered C_4_N_2_ ring is puckered as seen in the values of the puckering amplitude Q = 0.3609 (14) Å, θ = 63.3 (2) °, and φ = 324.4 (3) ° (Cremer & Pople, 1975); the respective values for the equivalent ring in molecule *b* are 0.3841 (14) Å, 63.2 (2) °, and 323.6 (2) °. This puckering results in a slightly folded conformation for the molecule, as indicated by the dihedral angle formed between the peripheral benzene rings of 12.64 (7) ° [11.72 (7) ° for molecule *b*].

Supramolecular arrays are found in the crystal structure of (I) mediated by N–H···N hydrogen bonding and sustained by C–H···N as well as C–H···π contacts, the latter involving hydrogen atoms from the methyl-C8 and -C24 groups and the ring centroids of the five-membered rings, Table 1 and Fig. 3. Layers stack along [0 1 1] as illustrated in Fig. 4, being associated via C–H···π contacts involving aromatic-H atoms and benzene rings.

### Experimental

Referring to Fig. 1, to a stirred solution of amine, 3, (10.75 mmol) in acetonitrile (43 ml) was bubbled hydrogen chloride gas. A precipitate was formed immediately. The resulting suspension was refluxed for 14 hours until homogeneous. The reaction mixture was evaporated at reduced pressure and the residue partitioned between CH_2_Cl_2_ and saturated aq. NaHCO_3_. The aqueous layer was washed with CH_2_Cl_2_, and the combined organic layers were dried over Na_2_SO_4_, filtered and concentrated under reduced pressure. To the solid residue, a mixture of amidine 4 and the starting aniline, 3, was added acetone (50 ml) and the mixture stirred for 30 min. and filtered. The filtrate was evaporated under reduced pressure to give 4 in 70% yield. Recrystallization from acetone of the insoluble residue from the filtration gave pale yellow blocks of (I); 5 in Fig. 1, in 15% yield. M. pt. 483-485 K.

### Refinement

The C-bound H atoms were geometrically placed (C–H = 0.95 Å) and refined as riding with U_iso_(H) = 1.2U_eq_(C). The N-bound H atoms were located from a difference map and refined with U_iso_(H) = 1.2U_eq_(N).

### Crystal data

**Table:**

C_16_H_14_ClN_3_	*Z* = 4
*M**_r_* = 283.75	*F*(000) = 592
Triclinic, *P*1	*D*_x_ = 1.412 Mg m^−^^3^
Hall symbol: -P 1	Mo *K*α radiation, λ = 0.71073 Å
*a* = 9.8636 (2) Å	Cell parameters from 5954 reflections
*b* = 10.7971 (2) Å	θ = 2.9–27.5°
*c* = 13.2387 (3) Å	µ = 0.28 mm^−^^1^
α = 93.483 (1)°	*T* = 120 K
β = 100.391 (1)°	Block, pale-yellow
γ = 104.419 (1)°	0.55 × 0.25 × 0.15 mm
*V* = 1334.81 (5) Å^3^	

### Data collection

**Table:**

Nonius KappaCCD area-detector diffractometer	6102 independent reflections
Radiation source: Enraf Nonius FR591 rotating anode	5108 reflections with *I* > 2σ(*I*)
10 cm confocal mirrors	*R*_int_ = 0.037
Detector resolution: 9.091 pixels mm^-1^	θ_max_ = 27.5°, θ_min_ = 2.9°
φ and ω scans	*h* = −12→12
Absorption correction: multi-scan (*SADABS*; Sheldrick, 2007)	*k* = −14→14
*T*_min_ = 0.885, *T*_max_ = 1.000	*l* = −17→17
27218 measured reflections	

### Refinement

**Table:**

Refinement on *F*^2^	Primary atom site location: structure-invariant direct methods
Least-squares matrix: full	Secondary atom site location: difference Fourier map
*R*[*F*^2^ > 2σ(*F*^2^)] = 0.036	Hydrogen site location: inferred from neighbouring sites
*wR*(*F*^2^) = 0.094	H atoms treated by a mixture of independent and constrained refinement
*S* = 1.02	*w* = 1/[σ^2^(*F*_o_^2^) + (0.0459*P*)^2^ + 0.5871*P*] where *P* = (*F*_o_^2^ + 2*F*_c_^2^)/3
6102 reflections	(Δ/σ)_max_ = 0.001
371 parameters	Δρ_max_ = 0.25 e Å^−^^3^
0 restraints	Δρ_min_ = −0.37 e Å^−^^3^

### Special details

**Table:**

Geometry. All s.u.'s (except the s.u. in the dihedral angle between two l.s. planes) are estimated using the full covariance matrix. The cell s.u.'s are taken into account individually in the estimation of s.u.'s in distances, angles and torsion angles; correlations between s.u.'s in cell parameters are only used when they are defined by crystal symmetry. An approximate (isotropic) treatment of cell s.u.'s is used for estimating s.u.'s involving l.s. planes.
Refinement. Refinement of *F*^2^ against ALL reflections. The weighted *R*-factor wR and goodness of fit *S* are based on *F*^2^, conventional *R*-factors *R* are based on *F*, with *F* set to zero for negative *F*^2^. The threshold expression of *F*^2^ > 2σ(*F*^2^) is used only for calculating *R*-factors(gt) etc. and is not relevant to the choice of reflections for refinement. *R*-factors based on *F*^2^ are statistically about twice as large as those based on *F*, and *R*- factors based on ALL data will be even larger.

### Fractional atomic coordinates and isotropic or equivalent isotropic displacement parameters (Å^2^)

**Table:**

	*x*	*y*	*z*	*U*_iso_*/*U*_eq_	
Cl1	0.33159 (4)	−0.34794 (3)	0.46107 (3)	0.02533 (10)	
N1	0.47189 (12)	0.14829 (11)	0.28749 (9)	0.0170 (2)	
H1N	0.5189 (18)	0.1492 (16)	0.2355 (13)	0.020*	
N2	0.30560 (12)	0.22108 (10)	0.36420 (8)	0.0146 (2)	
N3	0.25126 (12)	0.31785 (11)	0.39366 (9)	0.0166 (2)	
C1	0.37097 (15)	−0.20111 (13)	0.41036 (10)	0.0178 (3)	
C2	0.46556 (15)	−0.18303 (13)	0.34342 (11)	0.0191 (3)	
H2	0.5094	−0.2488	0.3275	0.023*	
C3	0.49562 (14)	−0.06863 (13)	0.30000 (11)	0.0180 (3)	
H3	0.5592	−0.0565	0.2532	0.022*	
C4	0.43335 (14)	0.02947 (13)	0.32429 (10)	0.0153 (3)	
C5	0.33861 (14)	0.01066 (13)	0.39313 (10)	0.0149 (3)	
C6	0.30674 (14)	−0.10637 (13)	0.43533 (10)	0.0166 (3)	
H6	0.2415	−0.1207	0.4808	0.020*	
C7	0.37046 (14)	0.22740 (13)	0.27162 (10)	0.0160 (3)	
C8	0.45339 (16)	0.36485 (14)	0.26594 (12)	0.0215 (3)	
H8A	0.5234	0.3969	0.3306	0.032*	
H8B	0.3871	0.4193	0.2555	0.032*	
H8C	0.5032	0.3670	0.2081	0.032*	
C9	0.25205 (16)	0.17480 (15)	0.17637 (11)	0.0231 (3)	
H9A	0.2939	0.1762	0.1146	0.035*	
H9B	0.1845	0.2282	0.1703	0.035*	
H9C	0.2017	0.0862	0.1831	0.035*	
C10	0.28291 (14)	0.11864 (13)	0.41894 (10)	0.0148 (3)	
C11	0.20930 (14)	0.15069 (13)	0.49425 (10)	0.0150 (3)	
C12	0.15553 (15)	0.09112 (13)	0.57641 (10)	0.0177 (3)	
H12	0.1643	0.0077	0.5897	0.021*	
C13	0.09056 (15)	0.15635 (14)	0.63631 (11)	0.0211 (3)	
H13	0.0535	0.1171	0.6915	0.025*	
C14	0.07742 (16)	0.28172 (14)	0.61761 (11)	0.0219 (3)	
H14	0.0322	0.3245	0.6608	0.026*	
C15	0.12848 (15)	0.34197 (13)	0.53880 (11)	0.0194 (3)	
H15	0.1199	0.4259	0.5271	0.023*	
C16	0.19440 (14)	0.27539 (13)	0.47525 (10)	0.0160 (3)	
Cl2	1.01515 (4)	0.86179 (3)	0.04959 (3)	0.02812 (11)	
N4	1.01094 (12)	0.36108 (11)	0.20762 (9)	0.0162 (2)	
H4N	1.0824 (18)	0.3620 (16)	0.2564 (13)	0.019*	
N5	0.76776 (12)	0.28013 (10)	0.13350 (8)	0.0147 (2)	
N6	0.64868 (12)	0.18074 (11)	0.10529 (9)	0.0159 (2)	
C17	1.01399 (15)	0.71382 (13)	0.09521 (11)	0.0182 (3)	
C18	1.13622 (15)	0.69989 (13)	0.15913 (11)	0.0186 (3)	
H18	1.2199	0.7697	0.1762	0.022*	
C19	1.13493 (14)	0.58330 (13)	0.19782 (10)	0.0171 (3)	
H19	1.2183	0.5733	0.2415	0.020*	
C20	1.01243 (14)	0.48057 (13)	0.17320 (10)	0.0146 (3)	
C21	0.88979 (14)	0.49500 (12)	0.10646 (10)	0.0143 (3)	
C22	0.89169 (15)	0.61293 (13)	0.06827 (10)	0.0166 (3)	
H22	0.8093	0.6239	0.0240	0.020*	
C23	0.87707 (14)	0.27850 (13)	0.22571 (10)	0.0157 (3)	
C24	0.89323 (16)	0.14258 (13)	0.23258 (12)	0.0209 (3)	
H24A	0.9129	0.1102	0.1675	0.031*	
H24B	0.8047	0.0865	0.2453	0.031*	
H24C	0.9725	0.1435	0.2894	0.031*	
C25	0.83271 (15)	0.33020 (15)	0.32098 (11)	0.0215 (3)	
H25A	0.9060	0.3329	0.3824	0.032*	
H25B	0.7414	0.2738	0.3288	0.032*	
H25C	0.8222	0.4172	0.3129	0.032*	
C26	0.76750 (14)	0.38327 (12)	0.08024 (10)	0.0142 (3)	
C27	0.63765 (14)	0.34888 (12)	0.00739 (10)	0.0146 (3)	
C28	0.57034 (15)	0.40825 (13)	−0.07201 (10)	0.0174 (3)	
H28	0.6155	0.4920	−0.0863	0.021*	
C29	0.43847 (15)	0.34197 (14)	−0.12786 (11)	0.0200 (3)	
H29	0.3911	0.3813	−0.1806	0.024*	
C30	0.37114 (15)	0.21577 (14)	−0.10858 (11)	0.0201 (3)	
H30	0.2795	0.1727	−0.1486	0.024*	
C31	0.43480 (15)	0.15426 (13)	−0.03367 (10)	0.0175 (3)	
H31	0.3900	0.0690	−0.0225	0.021*	
C32	0.56925 (14)	0.22232 (13)	0.02623 (10)	0.0155 (3)	

### Atomic displacement parameters (Å^2^)

**Table:**

	*U*^11^	*U*^22^	*U*^33^	*U*^12^	*U*^13^	*U*^23^
Cl1	0.0402 (2)	0.01712 (17)	0.02142 (18)	0.01136 (15)	0.00696 (15)	0.00566 (13)
N1	0.0154 (6)	0.0180 (6)	0.0202 (6)	0.0058 (5)	0.0075 (5)	0.0043 (5)
N2	0.0157 (5)	0.0143 (5)	0.0144 (5)	0.0050 (4)	0.0034 (4)	0.0015 (4)
N3	0.0191 (6)	0.0153 (6)	0.0165 (6)	0.0068 (5)	0.0041 (5)	0.0004 (4)
C1	0.0207 (7)	0.0149 (6)	0.0161 (6)	0.0053 (5)	−0.0011 (5)	0.0010 (5)
C2	0.0188 (7)	0.0192 (7)	0.0193 (7)	0.0090 (5)	−0.0001 (5)	−0.0019 (5)
C3	0.0149 (6)	0.0208 (7)	0.0183 (7)	0.0049 (5)	0.0041 (5)	−0.0006 (5)
C4	0.0130 (6)	0.0163 (6)	0.0150 (6)	0.0027 (5)	0.0007 (5)	0.0008 (5)
C5	0.0143 (6)	0.0156 (6)	0.0146 (6)	0.0053 (5)	0.0012 (5)	0.0005 (5)
C6	0.0172 (6)	0.0181 (7)	0.0145 (6)	0.0050 (5)	0.0022 (5)	0.0020 (5)
C7	0.0161 (6)	0.0191 (7)	0.0146 (6)	0.0060 (5)	0.0049 (5)	0.0036 (5)
C8	0.0244 (7)	0.0202 (7)	0.0233 (7)	0.0074 (6)	0.0093 (6)	0.0092 (6)
C9	0.0197 (7)	0.0350 (8)	0.0152 (7)	0.0100 (6)	0.0023 (6)	−0.0010 (6)
C10	0.0132 (6)	0.0155 (6)	0.0150 (6)	0.0031 (5)	0.0016 (5)	0.0022 (5)
C11	0.0129 (6)	0.0169 (6)	0.0144 (6)	0.0042 (5)	0.0009 (5)	0.0006 (5)
C12	0.0176 (7)	0.0190 (7)	0.0168 (7)	0.0052 (5)	0.0032 (5)	0.0040 (5)
C13	0.0199 (7)	0.0276 (8)	0.0161 (7)	0.0052 (6)	0.0055 (5)	0.0038 (6)
C14	0.0223 (7)	0.0258 (8)	0.0189 (7)	0.0093 (6)	0.0054 (6)	−0.0033 (6)
C15	0.0229 (7)	0.0175 (7)	0.0186 (7)	0.0085 (6)	0.0030 (6)	−0.0015 (5)
C16	0.0148 (6)	0.0170 (7)	0.0149 (6)	0.0040 (5)	0.0002 (5)	0.0005 (5)
Cl2	0.0325 (2)	0.01460 (17)	0.0300 (2)	−0.00067 (14)	−0.00427 (16)	0.00690 (14)
N4	0.0123 (5)	0.0167 (6)	0.0195 (6)	0.0039 (4)	0.0018 (5)	0.0055 (5)
N5	0.0147 (5)	0.0141 (5)	0.0146 (5)	0.0028 (4)	0.0029 (4)	0.0019 (4)
N6	0.0158 (5)	0.0138 (5)	0.0163 (6)	0.0007 (4)	0.0030 (4)	0.0004 (4)
C17	0.0221 (7)	0.0139 (6)	0.0180 (7)	0.0032 (5)	0.0044 (5)	0.0026 (5)
C18	0.0163 (7)	0.0179 (7)	0.0190 (7)	0.0001 (5)	0.0033 (5)	−0.0002 (5)
C19	0.0146 (6)	0.0196 (7)	0.0169 (7)	0.0049 (5)	0.0026 (5)	0.0016 (5)
C20	0.0167 (6)	0.0157 (6)	0.0132 (6)	0.0063 (5)	0.0049 (5)	0.0014 (5)
C21	0.0145 (6)	0.0143 (6)	0.0137 (6)	0.0026 (5)	0.0038 (5)	0.0007 (5)
C22	0.0174 (7)	0.0173 (7)	0.0149 (6)	0.0045 (5)	0.0023 (5)	0.0022 (5)
C23	0.0143 (6)	0.0167 (6)	0.0153 (6)	0.0034 (5)	0.0017 (5)	0.0034 (5)
C24	0.0209 (7)	0.0175 (7)	0.0244 (7)	0.0050 (6)	0.0030 (6)	0.0080 (6)
C25	0.0176 (7)	0.0295 (8)	0.0157 (7)	0.0046 (6)	0.0020 (5)	0.0004 (6)
C26	0.0159 (6)	0.0143 (6)	0.0138 (6)	0.0051 (5)	0.0049 (5)	0.0024 (5)
C27	0.0146 (6)	0.0157 (6)	0.0141 (6)	0.0043 (5)	0.0044 (5)	0.0010 (5)
C28	0.0193 (7)	0.0176 (7)	0.0163 (7)	0.0060 (5)	0.0042 (5)	0.0021 (5)
C29	0.0211 (7)	0.0247 (7)	0.0150 (7)	0.0096 (6)	0.0014 (5)	0.0004 (5)
C30	0.0148 (6)	0.0242 (7)	0.0182 (7)	0.0034 (5)	−0.0001 (5)	−0.0046 (6)
C31	0.0179 (7)	0.0158 (6)	0.0167 (7)	0.0012 (5)	0.0047 (5)	−0.0033 (5)
C32	0.0169 (6)	0.0160 (6)	0.0143 (6)	0.0045 (5)	0.0052 (5)	0.0005 (5)

### Geometric parameters (Å, °)

**Table:**

Cl1—C1	1.7411 (14)	Cl2—C17	1.7401 (14)
N1—C4	1.3884 (17)	N4—C20	1.3917 (17)
N1—C7	1.4649 (17)	N4—C23	1.4662 (17)
N1—H1N	0.896 (18)	N4—H4N	0.863 (17)
N2—C10	1.3532 (17)	N5—C26	1.3540 (17)
N2—N3	1.3550 (15)	N5—N6	1.3551 (15)
N2—C7	1.4791 (17)	N5—C23	1.4800 (17)
N3—C16	1.3573 (18)	N6—C32	1.3591 (17)
C1—C6	1.3835 (19)	C17—C22	1.3828 (19)
C1—C2	1.387 (2)	C17—C18	1.389 (2)
C2—C3	1.382 (2)	C18—C19	1.3855 (19)
C2—H2	0.9500	C18—H18	0.9500
C3—C4	1.3987 (19)	C19—C20	1.3939 (19)
C3—H3	0.9500	C19—H19	0.9500
C4—C5	1.4072 (19)	C20—C21	1.4118 (18)
C5—C6	1.3995 (19)	C21—C22	1.3954 (18)
C5—C10	1.4548 (18)	C21—C26	1.4506 (18)
C6—H6	0.9500	C22—H22	0.9500
C7—C8	1.5194 (19)	C23—C24	1.5214 (18)
C7—C9	1.5258 (19)	C23—C25	1.5261 (19)
C8—H8A	0.9800	C24—H24A	0.9800
C8—H8B	0.9800	C24—H24B	0.9800
C8—H8C	0.9800	C24—H24C	0.9800
C9—H9A	0.9800	C25—H25A	0.9800
C9—H9B	0.9800	C25—H25B	0.9800
C9—H9C	0.9800	C25—H25C	0.9800
C10—C11	1.4090 (19)	C26—C27	1.4080 (18)
C11—C12	1.4147 (19)	C27—C28	1.4137 (19)
C11—C16	1.4227 (18)	C27—C32	1.4240 (18)
C12—C13	1.370 (2)	C28—C29	1.370 (2)
C12—H12	0.9500	C28—H28	0.9500
C13—C14	1.424 (2)	C29—C30	1.420 (2)
C13—H13	0.9500	C29—H29	0.9500
C14—C15	1.369 (2)	C30—C31	1.372 (2)
C14—H14	0.9500	C30—H30	0.9500
C15—C16	1.4156 (19)	C31—C32	1.4114 (19)
C15—H15	0.9500	C31—H31	0.9500
			
C4—N1—C7	120.26 (11)	C20—N4—C23	119.47 (11)
C4—N1—H1N	114.6 (11)	C20—N4—H4N	114.1 (11)
C7—N1—H1N	111.3 (11)	C23—N4—H4N	111.7 (11)
C10—N2—N3	115.05 (11)	C26—N5—N6	114.95 (11)
C10—N2—C7	124.91 (11)	C26—N5—C23	124.25 (11)
N3—N2—C7	119.74 (11)	N6—N5—C23	120.24 (10)
N2—N3—C16	103.00 (11)	N5—N6—C32	103.14 (10)
C6—C1—C2	121.31 (13)	C22—C17—C18	121.23 (13)
C6—C1—Cl1	120.29 (11)	C22—C17—Cl2	119.69 (11)
C2—C1—Cl1	118.39 (10)	C18—C17—Cl2	119.08 (11)
C3—C2—C1	119.49 (13)	C19—C18—C17	119.40 (13)
C3—C2—H2	120.3	C19—C18—H18	120.3
C1—C2—H2	120.3	C17—C18—H18	120.3
C2—C3—C4	120.61 (13)	C18—C19—C20	120.59 (13)
C2—C3—H3	119.7	C18—C19—H19	119.7
C4—C3—H3	119.7	C20—C19—H19	119.7
N1—C4—C3	120.82 (12)	N4—C20—C19	121.43 (12)
N1—C4—C5	119.55 (12)	N4—C20—C21	118.93 (12)
C3—C4—C5	119.45 (12)	C19—C20—C21	119.51 (12)
C6—C5—C4	119.58 (12)	C22—C21—C20	119.53 (12)
C6—C5—C10	123.71 (12)	C22—C21—C26	123.40 (12)
C4—C5—C10	116.68 (12)	C20—C21—C26	117.06 (12)
C1—C6—C5	119.54 (13)	C17—C22—C21	119.71 (13)
C1—C6—H6	120.2	C17—C22—H22	120.1
C5—C6—H6	120.2	C21—C22—H22	120.1
N1—C7—N2	105.41 (10)	N4—C23—N5	105.27 (10)
N1—C7—C8	108.36 (11)	N4—C23—C24	108.66 (11)
N2—C7—C8	109.95 (11)	N5—C23—C24	109.72 (11)
N1—C7—C9	112.12 (11)	N4—C23—C25	112.27 (11)
N2—C7—C9	108.61 (11)	N5—C23—C25	108.52 (11)
C8—C7—C9	112.17 (12)	C24—C23—C25	112.16 (12)
C7—C8—H8A	109.5	C23—C24—H24A	109.5
C7—C8—H8B	109.5	C23—C24—H24B	109.5
H8A—C8—H8B	109.5	H24A—C24—H24B	109.5
C7—C8—H8C	109.5	C23—C24—H24C	109.5
H8A—C8—H8C	109.5	H24A—C24—H24C	109.5
H8B—C8—H8C	109.5	H24B—C24—H24C	109.5
C7—C9—H9A	109.5	C23—C25—H25A	109.5
C7—C9—H9B	109.5	C23—C25—H25B	109.5
H9A—C9—H9B	109.5	H25A—C25—H25B	109.5
C7—C9—H9C	109.5	C23—C25—H25C	109.5
H9A—C9—H9C	109.5	H25A—C25—H25C	109.5
H9B—C9—H9C	109.5	H25B—C25—H25C	109.5
N2—C10—C11	105.47 (11)	N5—C26—C27	105.54 (11)
N2—C10—C5	118.79 (12)	N5—C26—C21	118.71 (12)
C11—C10—C5	135.69 (13)	C27—C26—C21	135.74 (12)
C10—C11—C12	135.86 (13)	C26—C27—C28	135.70 (13)
C10—C11—C16	104.30 (12)	C26—C27—C32	104.40 (11)
C12—C11—C16	119.84 (12)	C28—C27—C32	119.90 (12)
C13—C12—C11	118.54 (13)	C29—C28—C27	118.35 (13)
C13—C12—H12	120.7	C29—C28—H28	120.8
C11—C12—H12	120.7	C27—C28—H28	120.8
C12—C13—C14	121.35 (13)	C28—C29—C30	121.35 (13)
C12—C13—H13	119.3	C28—C29—H29	119.3
C14—C13—H13	119.3	C30—C29—H29	119.3
C15—C14—C13	121.50 (13)	C31—C30—C29	121.81 (13)
C15—C14—H14	119.3	C31—C30—H30	119.1
C13—C14—H14	119.3	C29—C30—H30	119.1
C14—C15—C16	117.97 (13)	C30—C31—C32	117.58 (13)
C14—C15—H15	121.0	C30—C31—H31	121.2
C16—C15—H15	121.0	C32—C31—H31	121.2
N3—C16—C15	127.06 (13)	N6—C32—C31	127.08 (12)
N3—C16—C11	112.13 (12)	N6—C32—C27	111.93 (12)
C15—C16—C11	120.80 (13)	C31—C32—C27	120.99 (12)
			
C10—N2—N3—C16	2.18 (14)	C26—N5—N6—C32	2.02 (14)
C7—N2—N3—C16	176.26 (11)	C23—N5—N6—C32	173.79 (11)
C6—C1—C2—C3	−0.5 (2)	C22—C17—C18—C19	−0.9 (2)
Cl1—C1—C2—C3	178.29 (10)	Cl2—C17—C18—C19	178.41 (10)
C1—C2—C3—C4	1.0 (2)	C17—C18—C19—C20	0.0 (2)
C7—N1—C4—C3	152.54 (13)	C23—N4—C20—C19	150.56 (13)
C7—N1—C4—C5	−32.29 (18)	C23—N4—C20—C21	−33.59 (17)
C2—C3—C4—N1	174.78 (12)	C18—C19—C20—N4	177.17 (12)
C2—C3—C4—C5	−0.4 (2)	C18—C19—C20—C21	1.4 (2)
N1—C4—C5—C6	−175.99 (12)	N4—C20—C21—C22	−177.61 (12)
C3—C4—C5—C6	−0.75 (19)	C19—C20—C21—C22	−1.68 (19)
N1—C4—C5—C10	1.91 (18)	N4—C20—C21—C26	1.62 (18)
C3—C4—C5—C10	177.16 (12)	C19—C20—C21—C26	177.55 (12)
C2—C1—C6—C5	−0.6 (2)	C18—C17—C22—C21	0.6 (2)
Cl1—C1—C6—C5	−179.41 (10)	Cl2—C17—C22—C21	−178.75 (10)
C4—C5—C6—C1	1.3 (2)	C20—C21—C22—C17	0.7 (2)
C10—C5—C6—C1	−176.50 (12)	C26—C21—C22—C17	−178.46 (13)
C4—N1—C7—N2	44.02 (15)	C20—N4—C23—N5	46.66 (15)
C4—N1—C7—C8	161.69 (12)	C20—N4—C23—C24	164.13 (12)
C4—N1—C7—C9	−73.97 (16)	C20—N4—C23—C25	−71.22 (15)
C10—N2—C7—N1	−31.81 (16)	C26—N5—C23—N4	−34.21 (16)
N3—N2—C7—N1	154.73 (11)	N6—N5—C23—N4	154.83 (11)
C10—N2—C7—C8	−148.40 (13)	C26—N5—C23—C24	−150.96 (12)
N3—N2—C7—C8	38.14 (16)	N6—N5—C23—C24	38.07 (16)
C10—N2—C7—C9	88.52 (15)	C26—N5—C23—C25	86.17 (15)
N3—N2—C7—C9	−84.94 (14)	N6—N5—C23—C25	−84.79 (14)
N3—N2—C10—C11	−1.57 (15)	N6—N5—C26—C27	−1.68 (15)
C7—N2—C10—C11	−175.31 (11)	C23—N5—C26—C27	−173.07 (11)
N3—N2—C10—C5	−179.50 (11)	N6—N5—C26—C21	179.01 (11)
C7—N2—C10—C5	6.77 (19)	C23—N5—C26—C21	7.62 (19)
C6—C5—C10—N2	−171.72 (12)	C22—C21—C26—N5	−169.74 (12)
C4—C5—C10—N2	10.47 (18)	C20—C21—C26—N5	11.07 (18)
C6—C5—C10—C11	11.1 (2)	C22—C21—C26—C27	11.2 (2)
C4—C5—C10—C11	−166.67 (14)	C20—C21—C26—C27	−167.98 (14)
N2—C10—C11—C12	−178.65 (15)	N5—C26—C27—C28	−178.92 (15)
C5—C10—C11—C12	−1.3 (3)	C21—C26—C27—C28	0.2 (3)
N2—C10—C11—C16	0.26 (14)	N5—C26—C27—C32	0.58 (14)
C5—C10—C11—C16	177.66 (14)	C21—C26—C27—C32	179.71 (14)
C10—C11—C12—C13	178.45 (15)	C26—C27—C28—C29	−179.44 (14)
C16—C11—C12—C13	−0.33 (19)	C32—C27—C28—C29	1.13 (19)
C11—C12—C13—C14	−0.4 (2)	C27—C28—C29—C30	−1.2 (2)
C12—C13—C14—C15	0.3 (2)	C28—C29—C30—C31	−0.2 (2)
C13—C14—C15—C16	0.5 (2)	C29—C30—C31—C32	1.6 (2)
N2—N3—C16—C15	177.09 (13)	N5—N6—C32—C31	178.35 (13)
N2—N3—C16—C11	−1.92 (14)	N5—N6—C32—C27	−1.56 (14)
C14—C15—C16—N3	179.89 (13)	C30—C31—C32—N6	178.42 (13)
C14—C15—C16—C11	−1.2 (2)	C30—C31—C32—C27	−1.69 (19)
C10—C11—C16—N3	1.08 (15)	C26—C27—C32—N6	0.64 (15)
C12—C11—C16—N3	−179.79 (12)	C28—C27—C32—N6	−179.77 (12)
C10—C11—C16—C15	−178.01 (12)	C26—C27—C32—C31	−179.27 (12)
C12—C11—C16—C15	1.12 (19)	C28—C27—C32—C31	0.32 (19)

### Hydrogen-bond geometry (Å, °)

**Table:**

Cg1, Cg2, Cg3 and Cg4 are the centroids of the N2,N3,C10,C11,C16, N5,N6,C26,C27,C32, C1–C6 and C17–C22 rings, respectively.

**Table:**

*D*—H···*A*	*D*—H	H···*A*	*D*···*A*	*D*—H···*A*
N1—H1n···N6	0.896 (18)	2.319 (18)	3.2090 (17)	172.5 (15)
N4—H4n···N3^i^	0.863 (18)	2.390 (17)	3.2384 (17)	167.5 (15)
C9—H9b···N4^ii^	0.98	2.58	3.537 (2)	164
C25—H25b···N1	0.98	2.61	3.545 (2)	160
C24—H24c···Cg1^i^	0.98	2.90	3.8431 (17)	162
C8—H8c···Cg2	0.98	2.97	3.8929 (17)	157
C18—H18···Cg3^iii^	0.95	2.92	3.6630 (15)	135
C14—H14···Cg4^iv^	0.95	2.95	3.8062 (16)	151

Symmetry codes:  (i) *x*+1, *y*, *z*; (ii) *x*−1, *y*, *z*; (iii) *x*+1, *y*+1, *z*; (iv) −*x*+1, −*y*+1, −*z*+1.
